# Mechanisms of Exercise Capacity Improvement after Cardiac Rehabilitation Following Myocardial Infarction Assessed with Combined Stress Echocardiography and Cardiopulmonary Exercise Testing

**DOI:** 10.3390/jcm10184083

**Published:** 2021-09-09

**Authors:** Krzysztof Smarz, Tomasz Jaxa-Chamiec, Beata Zaborska, Maciej Tysarowski, Andrzej Budaj

**Affiliations:** 1 Centre of Postgraduate Medical Education, Department of Cardiology, Grochowski Hospital, 04-073 Warsaw, Poland; tomjch@kkcmkp.pl (T.J.-C.); zaborska@kkcmkp.pl (B.Z.); abudaj@kkcmkp.pl (A.B.); 2 Department of Cardiovascular Medicine, Hartford Hospital, University of Connecticut School of Medicine, Hartford, CT 06106, USA; mtysar@gmail.com

**Keywords:** cardiac rehabilitation, cardiopulmonary exercise testing, exercise capacity, stress echocardiography

## Abstract

Cardiac rehabilitation (CR) is indicated in all patients after acute myocardial infarction (AMI) to improve prognosis and exercise capacity (EC). Previous studies reported that up to a third of patients did not improve their EC after CR (non-responders). Our aim was to assess the cardiac and peripheral mechanisms of EC improvement after CR using combined exercise echocardiography and cardiopulmonary exercise testing (CPET-SE). The responders included patients with an improved EC assessed as a rise in peak oxygen uptake (VO_2_) ≥ 1 mL/kg/min. Peripheral oxygen extraction was calculated as arteriovenous oxygen difference (A-VO_2_Diff). Out of 41 patients (67% male, mean age 57.5 ± 10 years) after AMI with left ventricular ejection fraction (LVEF) ≥ 40%, 73% improved their EC. In responders, peak VO_2_ improved by 27% from 17.9 ± 5.2 mL/kg/min to 22.7 ± 5.1 mL/kg/min, *p* < 0.001, while non-responders had a non-significant 5% decrease in peak VO_2_. In the responder group, the peak exercise heart rate, early diastolic myocardial velocity at peak exercise, LVEF at rest and at peak exercise, and A-VO_2_Diff at peak exercise increased, the minute ventilation to carbon dioxide production slope decreased, but the stroke volume and cardiac index were unchanged after CR. Non-responders had no changes in assessed parameters. EC improvement after CR of patients with preserved LVEF after AMI is associated with an increased heart rate response and better peripheral oxygen extraction during exercise.

## 1. Introduction

Patients after acute myocardial infarction (AMI) entering cardiac rehabilitation (CR) often have a low exercise capacity (EC) and it is well established that a low EC is strongly associated with a poor prognosis [1,2,3,4]. In The Henry Ford Exercise Testing (FIT) Project, in patients with known coronary artery disease, EC was a strong predictor of mortality, myocardial infarction, and downstream revascularizations. Furthermore, patients with similar EC had an equivalent mortality risk, irrespective of the baseline revascularization status [5].

According to the current guidelines, comprehensive CR, including exercise training, dietary counseling, smoking cessation, risk factor modification, patient education, and psychosocial support with stress management, should be indicated in all patients after AMI [6]. In patients with AMI treated with percutaneous coronary intervention, CR based on aerobic exercise and strength training is safe and improves functional capacity, as well as the test duration, workload, and heart rate response [7,8,9]. In a large and representative community cohort of Dutch patients with the acute coronary syndrome, CR was associated with a survival benefit regardless of age, type of diagnosis, and type of intervention [10]. Evidence suggests that the mechanism of EC improvement in heart failure patients could be different in patients with a reduced and preserved left ventricular ejection fraction (LVEF). Central and peripheral mechanisms play a significant role in patients with a reduced LVEF, while, peripheral mechanisms play a significant role in patients with a preserved LVEF [11]. Moderate aerobic exercise training significantly improved the microvascular function of the lower extremities evidenced by functional magnetic resonance imaging in older adults [12]. However, the beneficial effects of CR may depend of the type of exercise. In a study of 124 healthy individuals, only aerobic endurance and high-intensive interval training, but not resistance training, were associated with increased telomerase activity and telomere length in mononuclear cells [13]. Previous studies reported that up to a third of patients that completed CR did not improve their EC due to exercise training performed at too low of an intensity [14] or due to chronotropic incompetence [15]. In a subanalysis of the Study on Aerobic INTerval EXercise training in coronary artery disease patients (SAINTEX-CAD), predictors of non-improvement were revealed as a higher baseline peak oxygen uptake (VO_2_) and oxygen uptake efficiency slope, history of elective percutaneous coronary intervention, older age, lower training intensity, and lower baseline physical activity [16]. A recent study revealed that routine exercise-based CR could not increase aerobic fitness probably due to the too low intensity of exercise prescribed [17]. Personalized exercise prescriptions are now recommended, but it is unclear which factors are responsible for EC improvement and how to individualize exercise training programs, to obtain the best results [18]. Simultaneously performed cardiopulmonary exercise testing and stress echocardiography (CPET-SE) is a useful tool to evaluate mechanisms of exercise intolerance in patients with heart failure and could also be helpful to investigate these mechanisms in patients after AMI [19,20,21,22,23].

## 2. Materials and Methods

### 2.1. Aim

The aim of this study was to assess cardiac and peripheral mechanisms of EC improvement after CR in patients after AMI without reduced LVEF using CPET-SE.

### 2.2. Study Population

Out of consecutive patients aged over 18 years treated for the first AMI between October 2015 and January 2019 and enrolled for EC assessment using CPET-SE [23], we recruited patients referred for CR. Study exclusion criteria were: previous AMI, history or presence of symptomatic congestive heart failure, permanent atrial fibrillation or atrial flutter, chronic obstructive pulmonary disease, heart surgery, peripheral nerve and musculoskeletal disorder, peripheral vascular disease with intermittent claudication, stroke with residual deficits, LVEF < 40% at least 4 weeks after AMI, residual coronary artery stenosis (>50%) after percutaneous coronary intervention, anemia (hemoglobin < 12 g/dL), decompensated thyroid disease, chronic kidney disease (creatinine clearance < 30 mL/min), severe valvular diseases, pulmonary hypertension, hypertrophic cardiomyopathy with left ventricular outflow tract obstruction, exercise-induced ischemia, pulmonary limitations of exercise, respiratory exchange ratio (RER) at peak exercise < 1.05, poor echocardiographic acoustic window, and lack of informed consent.

Educational level was classified as primary (elementary school or vocational certificate), secondary (upper secondary school), or tertiary (university degree). Self-assessed physical activity prior to AMI was categorized as low, moderate, or high according to International Physical Activity Questionnaire [24].

### 2.3. Combined Cardiopulmonary Exercise Testing and Stress Echocardiography

Symptom-limited cardiopulmonary exercise test and stress echocardiography were performed simultaneously with a Schiller Cardiovit CS-200 (Schiller, Baar, Switzerland) and an Ergo Spiro adapter (Ganshorn, Niederlauer, Germany) on semi-supine cycle ergometer eBike EL (ergoline GmbH, Bitz, Germany) and echocardiographic machine VIVID 9 (General Electric Medical System, Horten, Norway). In all cases, the ramp protocol with an incremental load of 12.5 watts/minute was used. Volumetric and gas calibration was performed daily before the tests. All patients were familiar with the exercise protocol and were encouraged to exercise at maximal effort (≥8 points using the 10-point Borg scale) [25]. During the stress test, we assessed the clinical and hemodynamic status of the patient, recorded electrocardiograms (12 leads), ventilation and gas exchange parameters. Peak VO_2_ (mL/kg/min) was averaged from the highest 20 s of exercise, the anaerobic threshold was calculated using a dual method approach. Peak VO_2_ in mL/kg/min was used as EC parameter.

Resting echocardiography was recorded before starting exercise. Peak exercise echocardiographic images were recorded at peak exercise, before effort termination. Left ventricular volumes were measured in 4- and 2-chamber apical views and LVEF was calculated using the modified Simpson’s rule [26]. Early mitral inflow velocity (E) was recorded in pulse-wave Doppler at the tip of mitral leaflets. Left ventricular systolic (s’) and early diastolic (e’) myocardial velocities were evaluated using pulsed-tissue Doppler and averaged from interventricular septum and lateral wall. Wall motion score index was calculated using 16-segment model. Stroke volume was calculated based on echocardiographic measurements as follows: stroke volume = 0.785 × left ventricular outflow tract diameter^2^ × velocity time integral. The arteriovenous oxygen difference (A-VO_2_Diff) was calculated using the Fick equation as follows: VO_2_/cardiac output calculated from echocardiography [19].

All cardiopulmonary and stress echocardiographic examinations were performed and interpreted by an experienced cardiologist according to the current recommendations [26,27,28,29]. Echocardiographic images were analyzed off-line using EchoPAC PC software v.110.0.x. Detailed description of the CPET-SE methodology was presented earlier [23].

### 2.4. Cardiac Rehabilitation

Patients participated in daily stationary CR lasting 3 weeks or 3 times per week 2-month-long ambulatory CR program during routine post-AMI treatment in regional centers.

The comprehensive cardiac rehabilitation program included education on topics related to cardiovascular diseases risk factors and their treatment, and advice for long-term secondary prevention as recommended in the guidelines [30]. In all patients, the CR program was based on medically supervised endurance exercise training supplemented by inspiratory muscles training. Intensity of exercise aerobic training sessions were specified based on maximal heart rate determined by symptom limited exercise testing [31].

During subsequent sessions, exercise training gradually increased to 50–60% or 60–80% of the heart rate reserve according to physicians’ decision. The training sessions consisted of cycling for about 60 min per session, including warming up and cooling down period.

Patients were divided into two groups: responders—who improved EC after CR—and non-responders—who did not improve EC. Improvement in EC was assessed as the difference in peak VO_2_ before and after CR ≥ 1 mL/kg/min [16,18,32].

### 2.5. Statistical Analysis

Data were presented as mean ± standard deviation or median and interquartile range (IQR; 25th–75th percentiles) for continuous variables or as a number (percentage) for categorical variables. Independent parameters were assessed using Student’s *t*-test and Kruskal–Wallis test for parametric values and chi-square test for categorical variables.

All statistical tests were two-sided. Statistical significance was established as *p* = 0.05, and all statistical analyses were performed using R statistical software version 3.6.1, R Foundation for Statistical Computing, Vienna, Austria.

## 3. Results

### 3.1. Baseline Characteristics

Out of 61 patients treated for AMI and referred for CR who had performed CPET-SE before and after CR, 20 patients were excluded because of submaximal effort in one of the examinations (RER < 1.05). Of the 41 patients enrolled in the study, 30 (73%) were responders. The daily beta-blocker dose was lower in the responder group, other baseline clinical characteristic parameters did not differ between groups. Clinical characteristics of studied population are presented in Table 1. All patients completed CR.

### 3.2. Combined Cardiopulmonary Exercise Testing and Stress Echocardiography before and after Cardiac Rehabilitation

#### 3.2.1. Cardiopulmonary Parameters

Cardiopulmonary exercise testing parameters are presented in Table 2. The overall peak VO_2_ increased by 17.5% from 18.4 ± 5.2 mL/kg/min to 21.7 ± 5.2 mL/kg/min, *p* = 0.006. In the responders, peak VO_2_ improved by 27% from 17.9 ± 5.2 mL/kg/min to 22.7 ± 5.1 mL/kg/min, *p* < 0.001, while non-responders had a non-significant 5% decrease in peak VO_2_. In responders, unlike non-responders, EC improvement was also seen as a percentage of predicted VO_2_ (68 ± 16% vs. 88 ± 19%, *p* < 0.0001, before and after CR, respectively). Responders also had improvement after CR in exercise time (407 ± 135 s vs. 491 ± 131 s, *p* = 0.016) and load achieved at peak exercise (98 ± 29 watts vs. 116 ± 28 watts, *p* = 0.017).

In the responder, but not in the non-responder group, the peak heart rate (108 ± 15 bpm vs. 116 ± 13 bpm, *p* = 0.027), percent predicted heart rate at peak exercise (67 ± 9% vs. 72 ± 8%, *p* = 0.029), and chronotropic index (42 ± 15% vs. 52 ± 12%, *p* = 0.005) improved after CR. The minute ventilation to carbon dioxide production slope (VE/VCO_2_ slope) (24 ± 5 vs. 21 ± 3, *p* = 0.025) decreased after CR in responders opposite to non-responders. Systolic blood pressure was higher at rest and at peak exercise after CR in the responder group; diastolic blood pressure slightly increased after CR (borderline statistical significance).

RER at peak exercise did not differ before and after CR in the responder (1.14 [IQR 1.07–1.25] vs. 1.14 [IQR 1.09–1.20], *p* = 0.480) and in the non-responder group (1.20 [IQR 1.12–1.22] vs. 1.17 [1.12–1.18], *p* = 0.598). There were no pulmonary limitations of exercise. None of the patients had breathing reserve ≤15% at peak exercise. Resting spirometry parameters were unchanged after CR in both groups. A-VO_2_Diff at peak exercise increased after CR in the responder group (13.9 ± 4.1 mL/dL vs. 17.0 ± 4.7 mL/dL, *p* = 0.009) but not in the non-responder group.

#### 3.2.2. Stress Echocardiography Parameters

Stress echocardiography parameters are presented in Table 3. In the responder group, unlike non-responders, LVEF at rest and at peak exercise was improved (57 [IQR 51–61]% vs. 62 [IQR 58–68]%, *p* = 0.002 and 64 [IQR 59–70]% vs. 73 [IQR 68–77]%, *p* = 0.001, for rest and peak exercise in the responder group). In the responder group, the left ventricular end-systolic volume was lower at rest and at peak exercise after CR. Left ventricular end-diastolic volume at peak exercise was lower after CR in the responder group. Only in the responder group the e’ at peak exercise increased after CR (12.6 ± 2.6 cm/s vs. 14.1 ± 2.5 cm/s, *p* = 0.024).

No differences in the left ventricular stroke volume, wall motion score index, and left ventricular systolic myocardial velocities before and after CR were noticed. The right ventricular systolic function was unchanged. There were no significant changes in mitral and tricuspid regurgitation before and after CR. None of the patients developed severe mitral or tricuspid regurgitation.

## 4. Discussion

**Figure 1:**
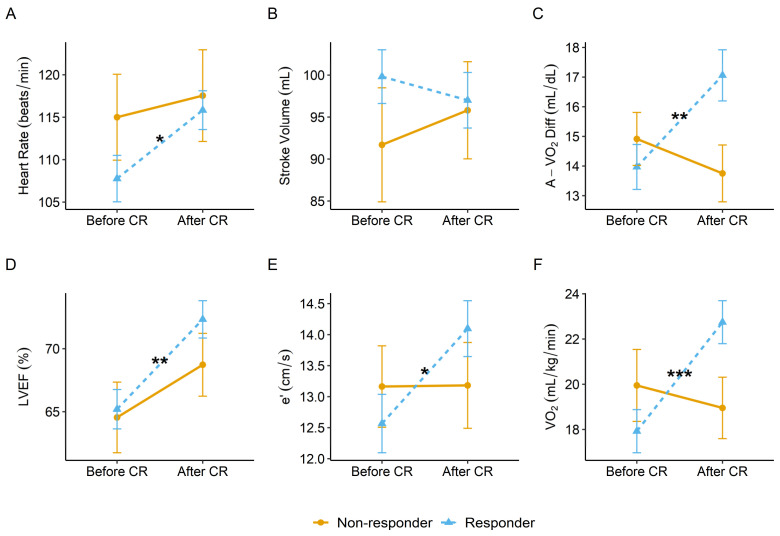
Peak exercise data before and after cardiac rehabilitation (CR) in responder and non-responder group for (**A**) heart rate, (**B**) stroke volume, (**C**) arteriovenous oxygen difference (A-VO_2_Diff), (**D**) left ventricular ejection fraction (LVEF), (**E**) early diastolic myocardial velocity (e’), and (**F**) oxygen uptake (VO_2_). Error bars represent standard error of the sample. * *p* = 0.03, ** *p* < 0.01, *** *p* < 0.001.

Our study revealed that post-AMI patients with LVEF ≥ 40%, who improved EC after CR, also improved heart rate response, peak exercise A-VO_2_Diff, peak exercise e’, and LVEF, but not stroke volume (Figure 1).

In a recently published study, we found that peak VO_2_ in post-AMI patients without reduced LVEF is related to chronotropic response and peripheral oxygen extraction [23] and, currently, we revealed that an improvement in these parameters contributes to peak VO_2_ improvement after CR.

To the best of our knowledge, our study is the first investigating mechanisms of EC improvement after CR in patients treated for AMI, using CPET-SE. Stress echocardiography allows assessing the cardiac function during exercise and complements information taken from cardiopulmonary exercise testing. CPET-SE also allows to non-invasively calculate A-VO_2_Diff as a marker of peripheral oxygen extraction by working skeletal muscles.

In our study, in 27% of patients, EC after CR did not improve. It was in accordance with previous studies which reported that up to 1/3 of patients failed to meaningfully improve their peak VO_2_ after CR, despite adequate compliance with training [15,16,18]. These patients presented a decrease or increase in peak VO_2_ within the test–retest variability EC (±6%) [32]. There was also evidence that patients who did not improve EC after CR could have a worse prognosis. In a study of 1171 patients with chronic coronary artery disease referred for CR after therapy for an acute coronary syndrome, coronary artery bypass grafting or a percutaneous coronary intervention, 23% of patients did not improve peak VO_2_ (non-responders). These patients had three-fold higher all-cause mortality in mean 6 years of follow-up as compared to responders, and a 1 mL/kg/min higher improvement in peak VO_2_ was associated with a 10% reduction in all-cause mortality [33].

Although some authors reported better EC in the non-responder group [16], in our study, differences in EC before CR were not significant (peak VO_2_ 17.9 ± 5.2 mL/kg/min vs. 19.9 ± 5.2 mL/kg/min, *p* = 0.729, % predicted VO_2_ 68 ± 16% vs. 74 ± 19% *p* = 0.335, for responders and non-responders, respectively).

The mechanisms and predisposing factors of this impaired response to CR are not fully recognized and understood. Potential factors influencing EC improvement after CR include cardiac and non-cardiac factors, comorbidities, but also the exercise dose and intensity and compliance to CR. Cardiac factors include chronotropic response to exercise, systolic and diastolic function, and non-cardiac factors include skeletal myopathy and disorders of the vascular, respiratory, and autonomic systems [18]. The significance of the above components can differ depending on the mechanisms underlying exercise impairment. In patients with heart failure and reduced LVEF, EC improvement is mostly related to improvement in the left ventricular contractility, but in patients with preserved LVEF to improvement in the left ventricular diastolic function and peripheral mechanisms [11,34,35].

We did not find significant differences between groups in educational levels. However, the lower educational levels could be associated with a lower socioeconomic status, higher prevalence of cardiovascular risk factors, and lower compliance. In a recently published multicenter study of patients referred for CR, peak VO_2_ was strongly associated with socioeconomic status assessed as the educational level and cardiovascular risk factors [36]. Our study was in concordance with previous studies that investigated the mechanisms of EC improvement after CR in patients with coronary artery disease. An invasive CPET study of 12 male patients (mean age, 47.8 years) with coronary artery disease revealed that mean peak VO_2_ increased by 22.5% (*p* < 0.0001) after 3 months of physical training. At rest and at submaximal exercise, heart rate, mean blood pressure, and cardiac output decreased after training, whereas stroke volume was unchanged and A-VO_2_Diff increased. The authors concluded that an increased maximal A-VO_2_Diff probably explains most of the increase in EC [37].

Peripheral mechanisms were studied previously and revealed that endurance training improves the endothelial function and skeletal muscle deoxygenation. In a study of 200 patients with coronary artery disease and LVEF >40%, the peripheral endothelial function assessed as flow-mediated dilatation of the brachial artery in ultrasound scanning improved independently of the mode of exercise training [38]. In another study of early post-AMI patients assigned to a CR group, aerobic training enhanced skeletal muscle deoxygenation assessed in near-infrared spectroscopy, and it was related to an increased EC [39].

In our study, the heart rate response during exercise improved after CR in the responder group. It was in accordance with previous studies. A meta-analysis of randomized trials of heart failure patients undergoing CR showed an average increase in peak heart rate of 4 beats/min (2.5%, *p* = 0.011) compared to the pre-training level [40]. In a study of 90 patients with ischemic heart disease and preserved LVEF (65% after acute coronary syndrome) referred for CR, only responders improved the chronotropic response assessed as a chronotropic index (45.1 ± 16.9% to 72.7 ± 34.1%, *p* < 0.01). Authors conclude that the positive adaptation of autonomic function takes place only in these patients who improve EC [41]. Impaired chronotropic competence was also a major predictor of poor training response in heart failure patients with sinus rhythm [15]. Endurance exercise training leads to favorable changes in chronotropic function related to balance between the sympathetic and parasympathetic autonomic nervous system [41]. A lack of improvement in chronotropic response after CR in non-responders could be associated with chronotropic incompetence, and also with a higher beta-blocker daily dose, but our sample size was too small to reveal these findings.

Although a resting left ventricular diastolic function in patients after AMI correlates with EC [42], there are scarce data regarding left ventricular diastolic function improvement after CR. In a study of 29 men with ST elevation AMI who received reperfusion therapy, from whom 15 were randomized to the CR group, the effect of an 8-week CR on diastolic function was investigated. Authors found that, compared to the baseline, patients in the training group had significant improvement in the functional capacity and maximum heart rate, but the left ventricular diastolic function did not change significantly after the CR [43]. Similarly, in another study of 86 patients in a training group after AMI, EC improvement was not related to the improvement of diastolic and systolic function [44].

In our study, in responders, the e’ improved at peak exercise, but this single parameter was not sufficient to assess diastolic function. None of studied patients had significant diastolic dysfunction during exercise measured as E/e’ ratio >14.

Although LVEF improved in responders after CR, the stroke volume was unchanged after CR. Improved LVEF could be explained by a lower left ventricular systolic volume as a result of a better left ventricular contractility.

Ventilatory efficiency assessed as a VE/VCO_2_ slope improved in the responder group. It is related to the direction and magnitude of change in the arterial carbon dioxide partial pressure and the fraction of the tidal volume to dead space ventilation [45]. Impaired ventilatory efficiency could reflect a higher left ventricular remodeling and neurohormonal activation [46]. Ventilatory efficiency could be improved after CR in patients after AMI as a result of an improved pulmonary and cardiac function during exercise [47].

Potential reasons of non-improvement in EC could include: a low intensity of exercise prescribed, inappropriate mode of training used, low compliance (compliance was not assessed in our study). The more personalized intensity of exercise, or high-intensity interval training, and strength training could produce better results.

Our study had several limitations as a consequence of patient preselection—only patients willing to participate in CR and capable to exercise with an adequate acoustic window were included. Additionally, as a consequence of the mode of exercise, cycle ergometer in a semi-recumbent position during CPET-SE and in an upright position during training sessions could cause lower extremity muscle fatigue in some untrained patients. Therefore, our results should not be directly translated into other types of physical activity. Furthermore, to assess peripheral oxygen extraction we calculated A-VO_2_Diff and, therefore, our results should not be directly compared with invasive studies. Our study was an observational study and the authors had no influence on the CR program. The CR program included aerobic endurance exercise training on a cycle ergometer and inspiratory muscles training. Resistance/strength training was not used for post-AMI patients; therefore, our results could differ from studies where strength training was used. The mode of exercise intensity calculation based on the heart rate reserve rather than on the VO_2_ reserve could be important for the effects of rehabilitation and peak VO_2_ improvement. Our results apply only to patients without a reduced LVEF. In patients with LVEF < 40%, other parameters, such as the left ventricular stroke volume, could play a significant role in EC improvement.

As our study was a single site study with a relatively small group of patients, our findings need to be confirmed in further prospective studies with a larger group of patients and also with various models of exercise training.

## 5. Conclusions

Our findings suggested that increased EC after CR of patients without a reduced LVEF after AMI is associated with an improvement in the heart rate response and peripheral oxygen extraction. The left ventricular systolic and diastolic function can also be improved after aerobic training, but its relation to improved EC is less clear.

CPET-SE is a valuable clinical tool for a CR results assessment and could be useful to individualize an exercise training program to optimize EC improvement.

## Figures and Tables

**Table 1:** Clinical characteristics of studied patients.

	All Patients (*n* = 41)	Responders (*n* = 30)	Non Responder (*n* = 11)	*p* Value
Demographics				
Male sex, *n* (%)	28 (66)	20 (67)	8 (73)	1
Age, years	57.5 ± 10	57.6 ± 10.0	57.3 ± 11.0	0.921
Body mass index, kg/m^2^	27.2 ± 4.2	27.6 ± 4.3	26.0 ± 3.9	0.284
Comorbidity, *n* (%)				
Current smoking	23 (56)	16 (53)	7 (64)	0.758
Hypertension	23 (56)	17 (57)	6 (55)	0.948
Hyperlipidemia	34 (83)	25 (83)	9 (82)	0.972
Diabetes mellitus/Impaired glucose tolerance	15 (37)	11 (37)	4 (36)	0.990
Educational stage, *n* (%)				
Primary	11 (27)	6 (20)	5 (45)	0.411
Secondary	21 (51)	16 (53)	5 (45)	0.960
Tertiary	9 (22)	8 (27)	1 (9)	0.569
Hospitalization during myocardial infarction				
STEMI, *n* (%)	18 (43)	12 (40)	6 (55)	0.611
Inferior	9 (22)	8 (27)	1 (9)	0.436
Lateral	6 (15)	5 (17)	1 (9)	0.913
Posterior	2 (5)	1 (3)	1 (9)	1
Anterior	6 (15)	3 (10)	3 (27)	0.375
NSTEMI, *n* (%)	23 (56)	18 (60)	5 (46)	0.634
Troponin T maximum plasma concentration, ng/L, * IQR	597 (165–2380)	574 (237–1975)	718 (156–3976)	0.805
Laboratory tests at discharge				
Hemoglobin, g/dL	14.0 ± 1.1	14.2 ± 1.2	13.7 ± 0.8	0.306
Creatinine clearance **, mL/min	110 ± 32	110 ± 35	109 ± 26	0.907
Physical activity before myocardial infarction, *n* (%)				
Small	9 (22)	6 (20)	3 (27)	0.942
Moderate	22 (54)	16 (53)	6 (55)	1
High	10 (24)	8 (27)	2 (18)	0.881
Cardiac rehabilitation				
Inpatient	20 (49)	14 (47)	6 (54)	0.795
Number of training sessions	20 ± 4	20 ± 4	20 ± 4	0.718
Patients with training target heart rate 50–60%	28 (68)	18 (60)	10 (91)	0.430
Patients with training target heart rate 60–80%	13 (32)	12 (40)	1 (9)	0.148
Time between CPET-SE before and after cardiac rehabilitation, days, * IQR	56 (47–88)	88 (56–129)	56 (47–88)	0.064
Medication during cardiac rehabilitation, *n* (%)				
ACE-I/ARB	38 (93)	28 (93)	10 (91)	0.958
Beta-blocker	33 (80)	24 (80)	9 (82)	0.965
Aspirin	41 (100)	30 (100)	11 (100)	1
Clopidogrel or ticagrelor	41 (100)	30 (100)	11 (100)	1
Statin	41 (100)	30 (100)	11 (100)	1
Calcium channel blocker	9 (22)	6 (20)	3 (27)	0.693
Diuretic	10 (24)	5 (17)	5 (45)	0.156
Beta-blocker daily dose, bisoprolol equivalent, mg	3.1 (1.8)	2.7 (1.3)	4.2 (2.7)	0.036

*Note:* Values represent mean ± SD, * median and interquartile range (IQR; 25th–75th percentiles) or number (%). ** Creatinine clearance calculated using the Cockroft–Gault equation. *Abbreviations:* ACE-I, angiotensin-converting enzyme inhibitors; ARB, angiotensin receptor blockers; STEMI, acute myocardial infarction with ST segment elevation; NSTEMI, acute myocardial infarction without ST segment elevation; CPET-SE, combined exercise echocardiography and cardiopulmonary exercise testing.

**Table 2:** Cardiopulmonary exercise testing parameters during CPET-SE before and after cardiac rehabilitation.

	Responders (*n* = 30)			Non-Responders (*n* = 11)		
	Before CR	After CR	*p* Value	Before CR	After CR	*p* Value
Exercise time, sec	407 ± 135	491 ± 131	0.016	436 ± 122	440 ± 111	0.939
Load max predicted, watts	151 ± 51	150 ± 49	0.922	150 ± 45	151 ± 45	0.948
Load peak, watts	98 ± 29	116 ± 28	0.017	101 ± 24	106 ± 23	0.607
VO_2_ max predicted, L/min	2.21 ± 0.74	2.19 ± 0.68	0.904	2.15 ± 0.62	2.18 ± 0.62	0.902
VO_2_ at peak, L/min	1.48 ± 0.52	1.87 ± 0.52	0.006	1.53 ± 0.34	1.50 ± 0.34	0.880
VO_2_ at peak, mL/kg/min	17.9 ± 5,2	22.7 ± 5.1	<0.001	19.9 ± 5,2	18.9 ± 4.5	0.639
% VO_2_ predicted, %	68 ± 16	88 ± 19	<0.0001	74 ± 19	72 ± 19	0.763
VO_2_ at AT, mL/kg/min	11.2 ± 3,0	13.0 ± 3.0	0.021	12.2 ± 3.5	11.8 ± 3.2	0.820
VCO_2_ at peak, L/min	1.75 ± 0.6	2.15 ± 0.60	0.012	1.80 ± 0.40	1.73 ± 0.35	0.669
RER at peak, * IQR	1.14 (1.07–1.25)	1.14 (1.09–1.20)	0.480	1.20 (1.12–1.22)	1.17 (1.12–1.18)	0.598
SBP at rest, mmHg	124 ± 15	130 ± 14	0.124	131 ± 15	131 ± 18	0.948
DBP at rest, mmHg	73.3 ± 8.1	77.5 ± 7.51	0.043	78.2 ± 8.4	71.82 ± 8.74	0.097
SBP at peak, mmHg	172 ± 22	186 ± 18	0.008	197 ± 21	189 ± 17	0.303
DBP at peak, mmHg	71 ± 13	66 ± 9	0.069	72 ± 14	72 ± 15	0.942
HR max predicted, bpm	163 ± 10	162 ± 10	0.927	163 ± 11	163 ± 11	0.984
HR at rest, bpm	68 ± 10	66 ± 11	0.397	69 ± 6	69 ± 7	0.869
HR at peak, bpm	108 ± 15	116 ± 13	0.027	115 ± 17	118 ± 18	0.734
% HR predicted, %	67 ± 9	72 ± 8	0.029	71 ± 7	72 ± 7	0.596
Chronotropic index, %	42 ± 15	52 ± 12	0.005	48 ± 12	51 ± 13	0.686
VE at peak, L/min	43.60 ± 11.60	50.18 ± 11.13	0.029	45.90 ± 7.40	44.78 ± 8.99	0.747
VT at peak, L	1.70 ± 0.50	1.93 ± 0.54	0.078	1.70 ± 0.50	1.58 ± 0.34	0.849
BR at peak, %	57 ± 11	52 ± 10	0.057	50 ± 11	53 ± 11	0.611
VE/VCO_2_ slope	24 ± 5	21 ± 3	0.025	25 ± 4	25 ± 4	0.896
IVC, L	2.97± 0.75	3.02 ± 0.57	0.768	2.98 ± 0.72	3.01 ± 0.80	0.925
% IVC predicted, %	75 ± 15	77 ± 15	0.673	73 ± 13	75 ± 19	0.813
FEV1, L	2.91 ± 0.69	3.00 ±0.67	0.649	2.70 ± 0.53	2.73 ± 0.53	0.812
% FEV1 predicted, %	94 ± 17	97 ± 14	0.560	86 ± 13	88 ± 17	0.775
FEV1/IVC, %	92 ± 17	96 ± 16	0.319	93 ± 10	94 ± 20	0.843
A-VO_2_Diff at rest, mL/dL	7.0 ± 2.7	7.5 ± 2.3	0.489	7.2 ± 2.7	7.0 ± 1.9	0.875
A-VO_2_Diff at peak, mL/dL	13.9 ± 4.1	17.0 ± 4.7	0.009	14.9 ± 2.9	13.7 ± 3.1	0.385

*Note:* Values represent mean ± SD, * median and interquartile range (IQR; 25th–75th percentiles) or number (%). *Abbreviations:* AT, anaerobic threshold; A-VO_2_Diff, arteriovenous oxygen difference; BR, breathing reserve; DBP, diastolic blood pressure; FEV 1, forced expiratory volume in the first second; HR, heart rate; IVC, inspiratory vital capacity; RER, respiratory exchange ratio; SBP, systolic blood pressure; VCO_2_, carbon dioxide production; VE, minute ventilation; VO_2_, oxygen uptake; VT, tidal volume.

**Table 3:** Stress echocardiography parameters of study participants before and after cardiac rehabilitation.

	Responders (*n* = 30)			Non-Responders (*n* = 11)		
	Before CR	After CR	*p* Value	Before CR	After CR	*p* Value
Rest						
LVOT diameter, cm	2.15 ± 0.15	2.15 ± 0.15	1	2.07 ± 0.18	2.07 ± 0.18	1
LVOT VTI, cm	22.3 ± 2.9	21.9 ± 3.0	0.657	23.2 ± 5.7	22.3 ± 5.2	0.704
Stroke volume, mL, * IQR	77 (73–89)	78 (71–84)	0.673	80 (61–86)	71 (62–79)	0.646
Cardiac output, L/min	5.51 ± 1.19	5.23 ± 1.18	0.368	5.39 ± 1.59	5.32 ± 1.91	0.924
Cardiac index, L/min/m^2^	2.67 ± 0.57	2.68 ± 0.58	0.346	2.79 ± 0.68	2.71 ± 0.85	0.807
WMSI, * IQR	1.12 (1.06–1.31)	1.06 (1.06–1.19)	0.142	1.19 (1.19–1.34)	1.06 (1.06–1.28)	0.154
LVEF, %, * IQR	57 (51–61)	62 (58–68)	0.002	58 (54–60)	64 (57–67)	0.188
LVEDV index, mL/m^2^	54 ± 13	49 ± 12	0.138	52 ± 16	50 ± 20	0.779
LVESV index, mL/m^2^	24± 9	19 ± 8	0.018	22 ± 10	20 ± 11	0.600
TAPSE, cm	2.2 ± 0.3	2.2 ± 0.3	0.943	2.2 ± 0.3	2.2 ± 0.3	0.784
RV s’, cm/s	12.2 ± 1.9	12.0 ± 1.9	0.775	11.3 ± 2.4	12.0 ± 1.7	0.421
LV s’, cm/s	8.0 ± 1.6	7.5 ± 1.3	0.132	7.9 ± 1.5	8.5 ± 1.6	0.375
e’, cm/s	9.0 ± 2.3	9.0 ± 2.5	0.949	9.4 ± 2.4	9.6 ± 2.9	0.864
E/e’ ratio	7.5 ± 2.3	6.9 ± 2.1	0.258	7.7 ± 2.4	7.8 ± 2.9	0.859
Mitral regurgitation, *n* (%) Mild/Moderate	14 (46)/1 (3)	20 (66)/1 (3)	0.410/1	7 (64)/0 (0)	7 (64)/0 (0)	1/-
Tricuspid regurgitation, *n* (%) Mild/Moderate	10 (33)/0 (0)	16 (53)/0 (0)	0.324/-	4 (36)/0 (0)	5 (45)/0 (0)	0.778/-
Peak exercise						
LVOT VTI, cm	27.5 ± 3.9	26.7 ± 3.7	0.421	26.9 ± 4.4	28.4 ± 5.0	0.489
Stroke volume, mL, * IQR	98 (86–115)	93 (86–104)	0.379	76 (74–109)	100 (81–110)	0.599
Cardiac output, L/min	10.80 ± 2.65	11.22 ± 2.37	0.517	10.49 ± 2.71	11.26 ± 2.76	0.517
Cardiac index, L/mL/m^2^	5.53 ± 1.40	5.76 ± 1.23	0.514	5.45 ± 1.23	5.78 ± 1.27	0.544
WMSI, * IQR	1.09 (1.06–1.31)	1.06 (1.06–1.12)	0.066	1.19 (1.06–1.28)	1.06 (1.06–1.19)	0.398
LVEF, %, * IQR	64 (59–70)	73 (68–77)	0.001	65 (58–68)	72 (66–74)	0.178
LV EDV index, mL/m^2^	53 ± 11	46 ± 9	0.013	52 ± 20	52 ± 21	0.992
LV ESV index, mL/m^2^	19 ± 7	13 ± 5	<0.001	20 ± 11	18 ± 13	0.699
TAPSE, cm	2.9 ± 0.4	2.9 ± 0.4	0.460	2.8 ± 0.8	3.0 ± 0.5	0.468
RV s’, cm/s	16.5 ± 3.1	16.8 ± 2.5	0.754	15.8 ± 2.5	16.2 ± 2.9	0.731
LV s’, cm/s	10.4 ± 1.6	10.8 ± 1.6	0.461	10.4 ± 1.9	10.9 ± 1.8	0.581
e’, cm/s	12.6 ± 2.6	14.1 ± 2.5	0.024	13.2 ± 2.2	13.2 ± 2.3	0.984
E/e’ ratio	7.9 ± 2.2	7.3 ± 2.0	0.263	7.6 ± 1.5	7.9 ± 1.7	0.642
Mitral regurgitation, *n* (%) Mild/Moderate	14 (47)/2 (7)	16 (53)/1 (3)	0.765/0.573	6 (54)/2 (18)	5 (45)/1 (1)	0.805/0.587
Tricuspid regurgitation, *n* (%) Mild/Moderate	10 (33)/0 (0)	14 (47)/0 (0)	0.489/-	4 (36)/1 (9)	7 (64)/0 (0)	0.458/-

*Note:* Values represent mean ± SD, * median and interquartile range (IQR; 25th–75th percentiles) or number (%). *Abbreviations:* E, early mitral inflow velocity; e’, early diastolic myocardial velocity; LVOT, left ventricular outflow tract; LVEF, left ventricular ejection fraction; LV EDV, left ventricular end-diastolic volume; LV ESV, left ventricular end-systolic volume; LV s’, left ventricular systolic myocardial velocity; RV s’, right ventricular systolic myocardial velocity; TAPSE, tricuspid annulus plane systolic excursion; WMSI, wall motion score index; VTI, velocity time integral.

## Data Availability

The complete raw dataset file was generated on 11 May 2021 and can be accessed via the Mendeley Data repository: https://data.mendeley.com/datasets/yn3yg6drss/1.
